# Real-Time Imaging of HIF-1α Stabilization and Degradation

**DOI:** 10.1371/journal.pone.0005077

**Published:** 2009-04-04

**Authors:** Ekaterina Moroz, Sean Carlin, Katerina Dyomina, Sean Burke, Howard T. Thaler, Ronald Blasberg, Inna Serganova

**Affiliations:** 1 Department of Neurology, Memorial Sloan-Kettering Cancer Center, New York, New York, United States of America; 2 Department of Medical Physics, Memorial Sloan-Kettering Cancer Center, New York, New York, United States of America; 3 Department of Radiology, Memorial Sloan-Kettering Cancer Center, New York, New York, United States of America; 4 Department of Epidemiology and Biostatistics, Memorial Sloan-Kettering Cancer Center, New York, New York, United States of America; Brunel University, United Kingdom

## Abstract

HIF-1α is overexpressed in many human cancers compared to normal tissues due to the interaction of a multiplicity of factors and pathways that reflect specific genetic alterations and extracellular stimuli. We developed two HIF-1α chimeric reporter systems, HIF-1α/FLuc and HIF-1α(ΔODDD)/FLuc, to investigate the tightly controlled level of HIF-1α protein in normal (NIH3T3 and HEK293) and glioma (U87) cells. These reporter systems provided an opportunity to investigate the degradation of HIF-1α in different cell lines, both in culture and in xenografts. Using immunofluorescence microscopy, we observed different patterns of subcellular localization of HIF-1α/FLuc fusion protein between normal cells and cancer cells; similar differences were observed for HIF-1α in non-transduced, wild-type cells. A dynamic cytoplasmic-nuclear exchange of the fusion protein and HIF-1α was observed in NIH3T3 and HEK293 cells under different conditions (normoxia, CoCl_2_ treatment and hypoxia). In contrast, U87 cells showed a more persistent nuclear localization pattern that was less affected by different growing conditions. Employing a kinetic model for protein degradation, we were able to distinguish two components of HIF-1α/FLuc protein degradation and quantify the half-life of HIF-1α fusion proteins. The rapid clearance component (t_1/2_ ∼4–6 min) was abolished by the hypoxia-mimetic CoCl_2,_ MG132 treatment and deletion of ODD domain, and reflects the oxygen/VHL-dependent degradation pathway. The slow clearance component (t_1/2_ ∼200 min) is consistent with other unidentified non-oxygen/VHL-dependent degradation pathways. Overall, the continuous bioluminescence readout of HIF-1α/FLuc stabilization *in vitro* and *in vivo* will facilitate the development and validation of therapeutics that affect the stability and accumulation of HIF-1α.

## Introduction

The HIF-1 (hypoxia inducible transcriptional factor 1) controls the expression of genes involved in critical aspects of cancer biology, such as angiogenesis, glucose metabolism, cell survival, invasion and tumor progression [1], [2], [3], [4], [5], [6], [7]. HIF-1 is a heterodimeric protein complex composed of two subunits: a stable and constitutively expressed HIF-1β, and an inducible, O_2_- and growth factor-regulated HIF-1α-subunit [8], [9]. HIF-1α protein is constantly modified posttranslationally by prolyl hydroxylases at Pro402 and/or Pro564 within the oxygen-dependent degradation (ODD) domain which promotes binding with pVHL (von Hippel-Lindau protein) and subsequent targeting for rapid proteasomal degradation. The half-life of HIF-1α protein, as determined by standard immunoblotting method, is about 5–8 min under normal oxygenated conditions [10], [11], [12]. It has been proposed, that interaction between pVHL and HIF-1α occurs in the nucleus, where HIF-1α protein is ubiquitinated and then exported to the cytoplasm for further proteasomal degradation [13]. Under hypoxic conditions, the prolyl hydroxylation reaction is inhibited and pVHL-HIF-1α interaction is abrogated, resulting in HIF-1α accumulation in the nucleus and dimerization with HIF-1β [14], [15]. The degradation of HIF-1α is also regulated in an O_2_-independent manner by the competitive binding to either heat shock protein 90 (HSP90), which stabilizes the protein [16], [17], or to the anchoring protein (RACK1), which leads to HIF-1α degradation by an oxygen-independent process [18].

HIF-1α is overexpressed in many human cancers compared to normal tissues [19], [20], and this overexpression is due to the interaction of a multiplicity of factors and pathways that reflect specific genetic alterations and extracellular stimuli (e.g., hypoxia) that impact on both synthesis and degradation [21]. The activation of oncogenes (H-ras and v-src) involving signaling cascades (PI3K and MAPK), as well as loss of function mutations in tumor suppressor genes (*VHL, PTEN* and *p53*), have also been shown to result in HIF-1α protein accumulation and increased expression of downstream HIF-1 target genes [9], [21], [22], [23], [24].

The objective of this study was to investigate and compare the tightly controlled level of HIF-1α protein in normal and tumor cells by imaging the dynamic response of different fusion variants of the *HIF-1α* gene linked to the *Firefly luciferase* (*FLuc*) gene. To accomplish this goal, we engineered several different reporter constructs and generated reporter cell lines to study the dynamic stability of HIF-1α under hypoxia and in the presence of a prolyl hydroxylation inhibitor - CoCl_2_, a protein synthesis inhibitor - cycloheximide, and a proteasomal degradation inhibitor - MG132. Using immunofluorescence microscopy, we observed different patterns of subcellular localization of HIF-1α/FLuc fusion protein between normal cells and cancer cells; similar differences were observed for HIF-1α in non-transduced, wild-type cells. By employing a kinetic model for protein degradation, we were able to quantify the half-life of HIF-1α/FLuc noninvasively in cells with different genetic backgrounds using a more sensitive dynamic bioluminescence imaging technique.

## Materials and Methods

### Generation of reporter vectors

**Figure 1:**
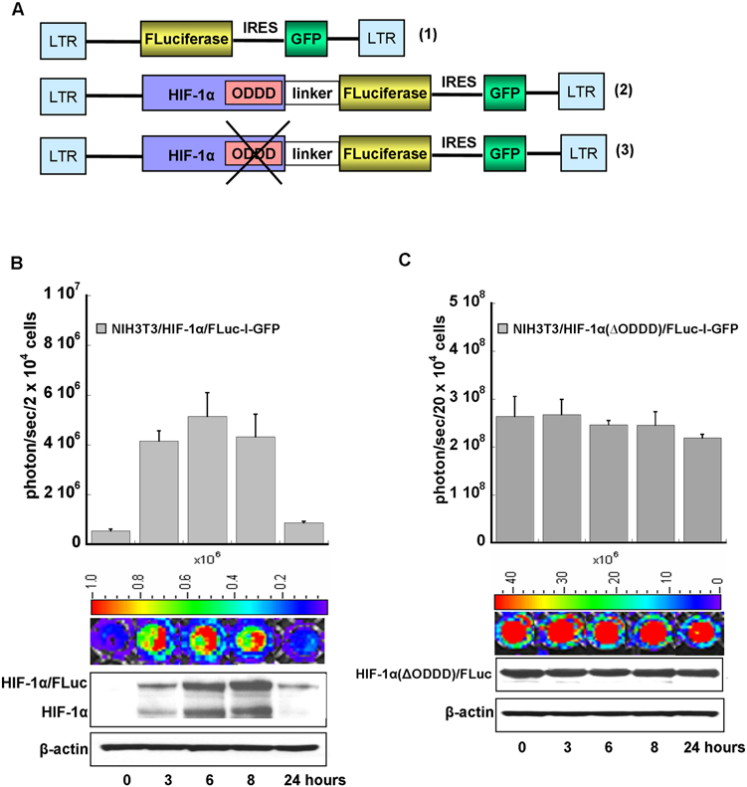
A. Structure of the reporter systems and development of the reporter cells. The retroviral plasmid with constitutively expressed Firefly Luicferase and GFP (1) was used as the backbone plasmid for developing the HIF-1α reporter systems and served as a control vector for Firefly Luciferase. Vector (2) contains a fusion between the full length cDNA of *HIF-1α and Firefly Luciferase* genes in a bicistronic cassette with IRES2-GFP. Vector (3) contains the fusion gene of *HIF-1α* with a deleted oxygen-dependent degradation domain (ODDD) sequence and *FLuciferase* in a bicistronic cassette with IRES2-GFP. B,C. Bioluminescence imaging of HIF-1α/Fluc accumulation in NIH3T3 reporter cells. Temporal changes in bioluminescence from HIF-1α/FLuc expression (B) and HIF-1α(ΔODDD)/Fluc expression (C) in NIH3T3 cells after treatment with CoCl_2_ (100 µM). Note the log-order difference in bioluminescence intensity (ordinate scale) between panels (B) and (C). NIH3T3 cells lysates were used for Western blotting with antibodies to HIF-1α to identify the HIF-1α/FLuc fusion protein and endogenous HIF-1α in the same cells.

All DNA manipulations were performed using restriction enzymes, T4 DNA ligase, CIP, and buffers according to standard procedures and manufacturer's instructions (Life Technologies, Inc., Roche and New England BioLabs, CA, USA). The initial retroviral vector SFG-FLuc-IRES2-GFP was developed through several steps using SFG-TK/GFP plasmid as a backbone [25] **(** **Fig. 1A** **(1)).** This vector contained constitutively expressed *FLuciferase* and *GFP (*green fluorescent protein) separated by an internal ribosome entry site (IRES2) [26]. Based on this plasmid, two additional retroviral vectors were developed: SFG-HIF-1α/FLuc-IRES2-GFP, SFG-HIF-1α(ΔODDD)/Fluc-IRES2-GFP **(** **Fig. 1A** **(2, 3)).**

To construct the SFG-HIF-1α/FLuc-IRES2-GFP vector, cDNA for HIF-1α was amplified from pCEP4-HIF-1α (ATCC, VA, USA) by 4 step PCR amplification with one primer for the 5′ end of HIF-1α cDNA that incorporated a *Nco*I restriction site: 5′-CGTCTTCCATGGATGCGGCTGCCTTTGCGGCTGCTTCCTTTGCGGCTGC and four consecutive primers for elongation of the PCR amplified fragment, and by introducing a sequence encoding for 30 amino acid linker downstream HIF-1α [27]: 5′-ACCATCCTCTAGAATGGAGGGCGCCGGCGGCGCGA, 5′-CGGCGGCTTCTGCAAGGTTAACTTGATCCAAAGCTCTGAGTAATTCTTC, 5′-CAGCTTCTTTCGCTGCGGCCCTCTTTTGCGGCGGCTTCTGCAAGGTTAAC 5′-GGCTGCTTCCTTTGCGGCTGCTTCCTTGGCTGCAGCTTCTTTCGCTGCG. The resulting PCR product was used for *Nco*I/*Xba*I ligation into the SFG-FLuc-IRES2-GFP backbone to obtain the final plasmid SFG-HIF-1α/FLuc-IRES2-GFP where the fusion gene *HIF-1α/FLuc* was separated from the *GFP* by an IRES element.

To construct a *HIF-1α/FLuc* fusion gene with a deleted oxygen-dependent degradation domain (ΔODDD), corresponding to 1206–1813 bp in the HIF-1α ORF and 401–603 amino acids the HIF-1α protein, the following approach was used. PCR primers, corresponding to the N-terminal and C–terminal ends of the HIF-1α protein and two additional primers corresponding to the internal part of protein without ODD domain were designed: 5′-AAAAGAAAAGTCTCGAGATGCAGC, 5′-ATAGTTTAGCGGCCGCAGCAAAGTTAA, 5′-CATAGAAGCGGCCGCAGACTCAAATAC, 5′-GGCGGATCCTTACACGGCGATCT. PCR products with *Xho*I/*Not*I and *Not*I/*Bam*HI ends were subcloned into the SFG-HIF-1α/Fluc-IRES2-GFP between *Xho*I and *Bam*HI sites to generate the *HIF-1α(*Δ*ODDD)/FLuc* fusion gene without the ODDD sequence.

SFG-FLuc-IRES2-GFP, SFG-HIF-1α/FLuc-IRES2-GFP, SFG-HIF-1α(ΔODDD)/Fluc-IRES2-GFP retroviral plasmids were transfected into a GPG293 producer cell line using LipofectAMINE 2000 (Invitrogen, CA)[28] according to the manufacturer's protocol. The retrovirus-containing medium was collected over four consecutive days and stored at −80°C.

### Cells transduction

All cell lines were stably transduced by incubating 50% confluent cell cultures with a virus-containing medium for 12 hours in presence of polybrene (8 mg/ml; Sigma, St.Louis, MO, USA). Cells were sorted using the fluorescence-activated cell sorter (FACS; BD Bioscience, CA, USA) with identical gates to obtain populations of cells with the same GFP fluorescence level **(Fig. S1**).

### *In vitro* bioluminescence assays

Stably transduced and sorted cells were seeded in 6-well plates. The medium was changed 24 h later with fresh medium containing CoCl_2_ (100 µM) (Sigma-Aldrich, St. Louis, MO, USA). Alternatively cells were incubated under hypoxic conditions (2.5% O_2_) during 3, 6, 8 and 24 h in a HERA cell 150 chamber (Thermo Electron Corporation, USA). The cells were collected in PBS with 10% FCS, counted with a disposable hemocytometer (Incyto, SKC Co., Republic of Korea), and assessed for viability by trypan blue staining. Bioluminescence assays were always performed on 2×10^4^ cells in 96-well plates using 10 µl of Bright-Glo Luciferase solution (Promega Cor., Madison, WI, USA). An IVIS® Imaging System 200 (Caliper Life Sciences, CA) and Photon imager (Biospace, Paris, France) were used to measure FLuciferase activity. The acquisition time was dependent on the signal intensity in the different reporter cell lines. All measurements are reported as photons/second/2×10^4^ cells, folds or % of FLuciferase activity.

### Protein degradation assay and half-life calculation

Cells were seeded into 96-well plates. After 3–4 hours of incubation in standard conditions, the protein synthesis inhibitor cycloheximide (100 µg/ml) (Sigma-Aldrich, St. Louis, MO, USA) was added for the indicated periods of time to the medium. In the experiments with CoCl_2_ treatment, cells were pretreated with CoCl_2_ for 3 h which was either maintained in the medium during cycloheximide exposure or was removed before exposure to cycloheximide. Following incubation, the cells were assayed for bioluminescence using Bright-Glo Luciferase solution. FLuciferase activity of the test cells was normalized to that of untreated control cells and expressed as % of control. The times profiles for FLuciferase activity were generated and fitted to either a single or double exponential equation to estimate the degradation rate of FLuicferase activity (reflecting the amount of HIF-1α/FLuc fusion protein): % of control = A_0_·e∧(−k_a_·t) or A_0_·e∧(−k_a_·t)+B_0_·e∧(−k_b_·t), respectively. The half-life was calculated by: t_1/2_ = ln2/k. Data fitting was performed using KaleidaGraph (Synergy Software version 3.6.4).

### Western blotting

Cell lines underwent protein extraction using Mammalian Protein Extraction Reagent (Pierce, Rockford, IL, USA) or SDS sample buffer (62.5 mM Tris-HCl/pH 6.8, 2% w/v SDS, 10% Glycerol, 50 mM DTT). Protein concentrations were determined by Bio-Rad protein assay (Bio-Rad, Hercules, CA, USA). The proteins in equivalent amounts (10–40 µg/well) were separated by electrophoresis in a NuPAGE gradient 4–12% Bis-Tris Gel (Invitrogen, Carlsbad, CA, USA) and were immuno-blotted with anti-HIF-1α (RD Systems, Minneapolis, MN, USA) at a 1∶200 dilution, and anti-ß-actin ( Abcam Inc., Cambridge, MA, USA ) at a 1∶5,000 dilution antibodies. Immune complexes were detected by horseradish-peroxidase-labeled antibodies and enhanced chemiluminescence reagent (Amersham, Buckinghamshire, UK).

### Immunofluorescence microscopy

Adherent cells were fixed with 4% paraformaldehyde in PBS for 12 min, washed three times with PBS, permeabilized with 0.2% Triton X-100 in Superblock (Pierce, Rockford, Il) for 30 min and washed again with PBS (at room temperature). After blocking nonspecific binding with ImageIT (Molecular Probes, Carlsbad, CA) for 30 min at room temperature, the cells were incubated with mouse anti-HIF-1α (BD Biosciences, San Jose, CA and RD Systems, Minneapolis, MN, USA) at 1∶50 dilution in Superblock overnight at 4°C. Primary antibodies were detected by incubation with a goat anti-mouse secondary antibody conjugated to Alexa-568 (Molecular Probes Carlsbad, CA) diluted in Superblock at 1∶100, at room temperature for 1 h. Following washing in PBS, cells were mounted in Vectashield (Burlingame, CA) containing 1.5 mg/ml DAPI. Fluorescence images were acquired at ×40 magnification using an Olympus BX40 fluorescence microscope (Olympus America, Inc.) with CCD camera. DAPI, GFP and HIF-1α were imaged using blue, green and red filters respectively.

### *In vivo* bioluminescence imaging

The animal protocol was approved by the Memorial Sloan-Kettering Institutional Animal Care and Use Committee. The U87/HIF-1α/Fluc, U87/HIF-1α(ΔODDD)/Fluc and U87/Fluc reporter cells (4×10^6^) were injected subcutaneously onto the right shoulder of 6-weeks old male Ncr *nu/nu* nude mice. After 1 week of tumor growth, a size of the xenografts reached a diameter of ∼6–7 mm. For the FLuciferase substrate delivery into mice, the micro-osmotic pumps (Alzet Model 1007D) (Durect Corporation, CA, USA), loaded with D-Luciferin (50 mg/ml) (Caliper Life Sciences, CA, USA), were surgically implanted in the dorsal back subcutaneously. Mice were allowed to recover for 48 h and than imaged using IVIS imaging system before and after intraperitoneal injection of CoCl_2_ (60 mg/kg). Photons emitted from the tumor region were quantified using Living Image software (Caliper Life Sciences, CA).

### Statistical analyzes

Each experiment or assay was performed at least three times in triplicate and representative examples are shown. Data are reported as means±SD, applying Dunnett's ANOVA-based multiple comparisons procedure for comparing several treatments (CoCl_2_ and hypoxia) with a single control and separately for each reporter. P<0.05 is considered significant.

## Results

### Development of reporter cells

Three new retroviral vectors were developed to study the regulation of HIF-1α accumulation/degradation processes in cells**.** Fusion genes containing *HIF-1α* ( **Fig. 1A** **(2)**), or a mutant variant of the *HIF-1α (*Δ*ODDD)* with a deleted oxygen-dependent degradation domain (**Fig. 1A** **(3)**), were linked to the *Firefly Luciferase* by a rationally designed linker of 30 amino acids [27]. *GFP,* separated by an IRES2 element in the fusion gene cassettes, was used to FACS-sort GFP-positive transduced cells in order to obtain populations of cells with similar GFP expression levels (**Fig. S1**). NIH3T3 (immortalized mouse fibroblasts) and U87 (human glioblastoma) cells were stably transduced with these reporter vectors. The reporter cell lines included: NIH3T3/HIF1α/FLuc, U87/HIF1α/FLuc, as well as NIH3T3/HIF-1α(ΔODDD)/Fluc, U87/HIF-1α(ΔODDD)/Fluc, and control NIH3T3/FLuc, and U87/FLuc. We also transduced HEK293 (human embryonic kidney) cells with the SGF-HIF1α/FLuc-IRES2-GFP reporter system.

### Bioluminescence imaging of HIF-1α levels in cells

To characterize the reporter systems, we performed several experiments to show the functionality of the HIF1α/FLuc and HIF-1α(ΔODDD)/FLuc fusion proteins in NIH3T3 reporter cells. Bioluminescence intensity was measured following the addition of an inhibitor of prolyl hydroxylation, a hypoxia mimetic, CoCl_2_ (100 µM), to the culture medium (**Fig. 1B, C**). An increase of HIF-1α/FLuc expression was detectable within the first several hours, reaching a maximum value within 3–8 h, followed by a return toward basal levels following 24 h of treatment. These data were confirmed by Western blotting for the fusion HIF-1α/FLuc and for endogenous HIF-1α proteins (**Fig. 1B**). In the case of reporter cells bearing the HIF-1α(ΔODDD)/FLuc fusion, we did not see any significant changes in bioluminescence signal over 24 h as well as in the fusion protein (**Fig. 1C**).

### Imaging HIF-1α levels in different cell lines

**Figure 2:**
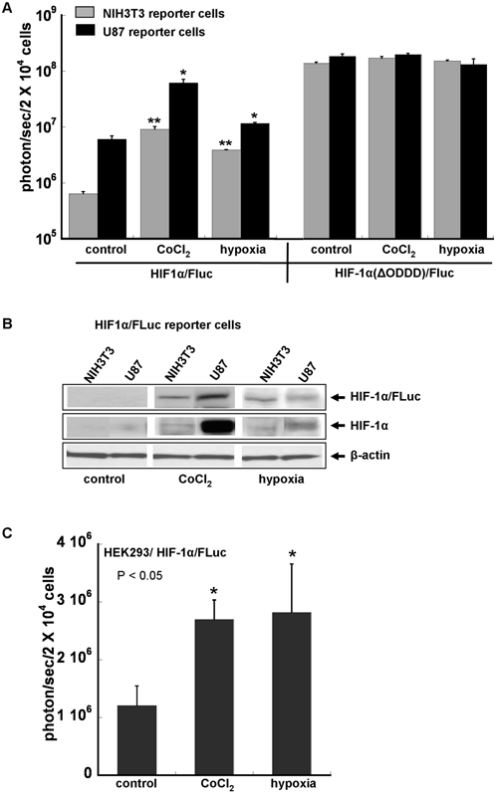
Comparison of HIF-1α/Fluc levels in NIH3T3, HEK293 and U87 cell lines. A comparison of HIF-1α/FLuc and HIF-1α(ΔODDD)/Fluc bioluminescence after treatment with CoCl_2_ (100 µM) and under low oxygen concentration (2.5% O_2_) for 6 h in NIH3T3 and U87 reporter cells. *: p<0.01 vs. the control. **: p<0.0001 vs. the control (A). The level of the expression HIF-1α/FLuc reporter and endogenous HIF-1α in reporter cells under the same conditions (B). Bioluminescence imaging of HEK293/HIF-1α/Fluc cells after treatment with CoCl_2_ (100 µM) and under low oxygen concentration (2.5% O_2_) for 6 h (C).

The HIF-1α/FLuc and HIF-1α(ΔODDD)/Fluc fusion proteins expression in NIH3T3 fibroblasts was compared to that obtained in reporter-transduced U87 glioma cells (**Fig. 2A**). The HIF1α/FLuc basal expression under normoxia, as measured by bioluminescence intensity, was approximately 9.5-fold higher (p = 0.006) in reporter-transduced U87 cells than in the reporter-transduced NIH3T3 cells (**Fig. 2A**). Exposing the NIH3T3 reporter-transduced cells to 100 µM CoCl_2_ resulted in a ∼15-fold increase (p<0.0001) in bioluminescence (**Fig. 2A**). Moderate hypoxia (2.5% O_2_) was less effective than CoCl_2_ in these cells, but still resulted in a statistically significant 6-fold increase (p<0.0001) in bioluminescence signal intensity. The U87/HIF1α/FLuc cells demonstrated a ∼10-fold (p<0.0001) and ∼2-fold (p = 0.0016) increase in bioluminescence in response to CoCl_2_ treatment and hypoxia, respectively **(** **Fig. 2A**). The HEK293/HIF1α/FLuc reporter cells as well showed low normoxic baseline levels of bioluminescence and an increase in bioluminescence following exposure to hypoxia (2.5% O_2_) and CoCl_2_ (100 µM) (**Fig. 2C** **)**.

We also measured bioluminescence of cells transduced with a mutant-variant of HIF-1α, containing a deleted oxygen-dependent degradation domain (ΔODDD) in the fusion construct (**Fig. 2A**). As expected, bioluminescence activity from NIH3T3 and U87 HIF-1α(ΔODDD)/Fluc reporter cells under baseline normoxic conditions was much higher than that in cells transduced with the HIF-1α/Fluc fusion. The bioluminescence signal from the NIH3T3/HIF-1α(ΔODDD)/Fluc reporter cells under normoxia was ∼300-fold greater (p<0.0001) than that of HIF-1α/Fluc transduced cells. A similar comparison of U87 reporter cells demonstrated a ∼50-fold increase (p = 0.0002) in bioluminescence signal as a result of ODDD deletion. The expression levels of the mutated HIF-1α(ΔODDD)/Fluc reporter in NIH3T3 and U87 cells were similar to each other and not influenced by treatment with CoCl_2_ or hypoxia (**Fig. 2A**).

To examine whether the increase in bioluminescence was due to HIF-1α stabilization or to an effect of CoCl_2_ on the FLuc reporter, the same experiments were performed using cells constitutively expressing Firefly Luciferase. FLuc bioluminescence intensity was not influenced by exposure to 100 µM CoCl_2_ or hypoxia (2.5% O_2_) (data not shown). All bioluminescence data were confirmed by Western blotting using specific antibodies (**Fig. 2B**).

### The analysis of HIF-1α distribution in cells by immunofluorescence microscopy

**Figure 3:**
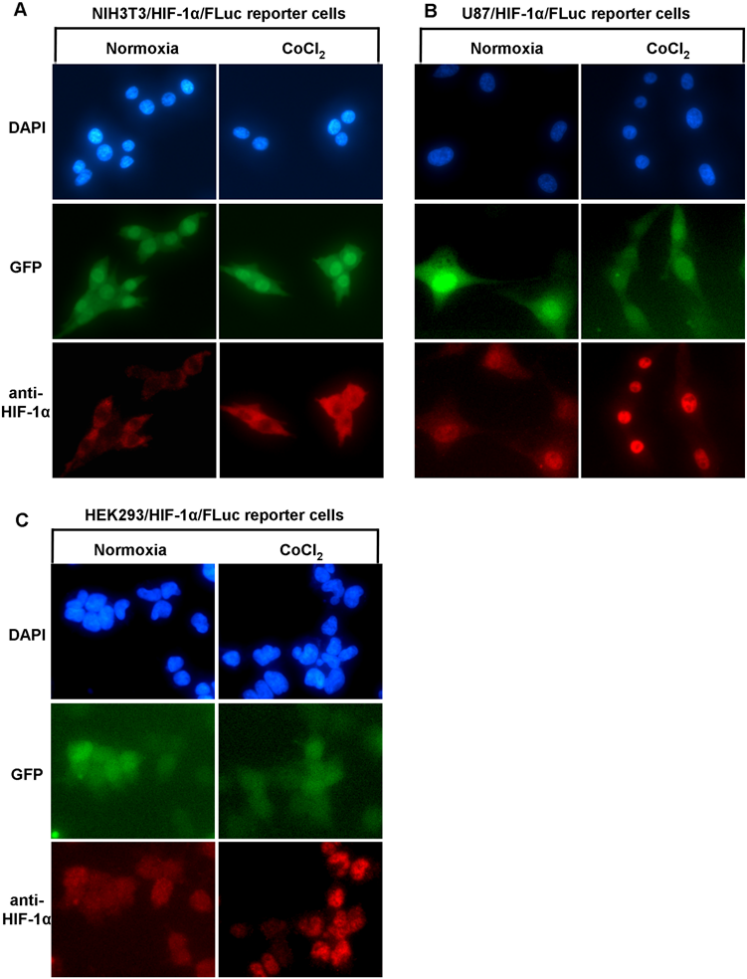
Immunofluorescence analysis of HIF-1α/Fluc trafficking in reporter NIH3T3, HEK293 and U87 cell lines. The NIH3T3/HIF-1α/Fluc (A), U87/HIF-1α/Fluc (B) and HEK293/HIF-1α/Fluc (C) reporter-transduced cells were cultured under normoxic conditions or in the presence of CoCl_2_ (100 µM) for 6 h. Cells were prepared for immunofluorescence as described in Materials and Methods and were incubated with the anti-HIF-1α antibody followed by an Alexa-568-conjugated secondary antibody. Cells were also visualized for GFP expression. Fluorescence images were acquired using the same acquisition parameters at ×40 magnification. All panels represent the magnified images after contrast and brightness adjustment to visualize the subcellular localization of the protein.

**Figure 4:**
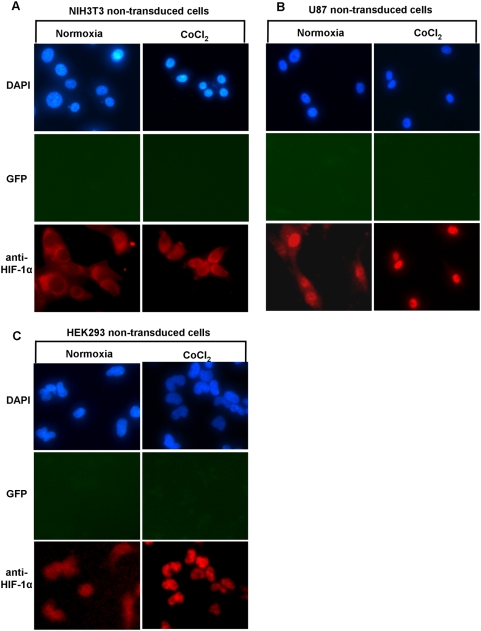
Immunofluorescence analysis of endogenous HIF-1α trafficking in wild-type NIH3T3, HEK293 and U87 cell lines. The non-transduced wild-type NIH3T3 (A), U87 (B) and HEK293 (C) cells were cultured under normoxic conditions or in the presence of CoCl_2_ (100 µM) for 6 h. Cells were prepared for immunofluorescence as described in Materials and Methods and were incubated with the anti-HIF-1α antibody followed by an Alexa-568-conjugated secondary antibody. Cells were also visualized for GFP expression. Fluorescence images were acquired using the same acquisition parameters at ×40 magnification. All panels represent the magnified images after contrast and brightness adjustment to visualize the subcellular localization of the protein.

**Figure 5:**
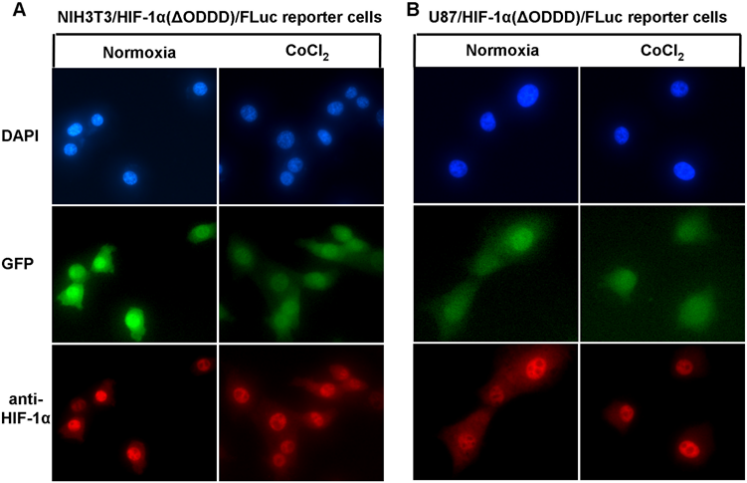
Immunofluorescence analysis of HIF-1α(ΔODDD)/Fluc trafficking in NIH3T3 and U87 reporter cell lines. NIH3T3/HIF-1α(ΔODDD)/Fluc and U87/HIF-1α(ΔODDD)/Fluc reporter-transduced cells were cultured under normoxic conditions or in the presence of CoCl_2_ (100 µM) for 6 h. Cells were prepared for immunofluorescence and analyzed as described in the legend to Figure 3.

Having characterized the behavior of the reporter proteins in the stably transduced cell lines, we sought to investigate whether the different genetic backgrounds of the cells influenced the intracellular localization of HIF-1α and its fusion proteins. To show that endogenous HIF-1α protein possess the same cytoplasmic-nuclear trafficking patterns as the HIF-1α/FLuc reporter, we also performed immunofluorescence staining of non-transduced, wild-type NIH3T3, U87 and HEK293 cells. As seen by immunofluorescence microscopy under normoxia HIF-1α/FLuc localization in NIH3T3 reporter cells (**Fig. 3A** **)** and HIF-1α in wild-type NIH3T3 cells (**Fig. 4A**) was primarily cytoplasmic, compared to the more equal distribution between nucleus and cytoplasm in HEK293 reporter and wild-type cells (**Fig. 3C** **,** **4C**). In contrast, more intense nuclear staining of HIF-1α/FLuc (**Fig. 3B**) and wild-type HIF-1α (**Fig. 4B**) was observed in U87 reporter and non-transduced cells, respectively. Following exposure to CoCl_2_ there was a consistent shift in HIF-1α/FLuc and HIF-1α localization to the nucleus in all three cell lines. NIH3T3 reporter and wild-type cells showed increased accumulation in both compartments, with more intense cytoplasmic accumulation **(** **Fig. 3A** **,** **4A** **).** In contrast, U87 and HEK293 reporter-transduced and wild-type cells showed much greater accumulation of HIF-1α/FLuc and HIF-1α in the nucleus following CoCl_2_ treatment **(** **Fig. 3B, 3C** **,** **4B, 4C** **)**. The same pattern of the HIF-1α/FLuc and HIF-1α re-distribution was observed after exposure of all three reporter-transduced cell lines to hypoxia (2.5% O_2_) **(Fig. S2, S3, S4)**. Interestingly, the deletion of the oxygen-dependent degradation domain from HIF-1α resulted in a predominant nuclear accumulation of the mutant fusion protein under normal growth conditions, hypoxia (2.5% O_2_) or CoCl_2_ treatment, for both NIH3T3 and U87 HIF-1α(ΔODDD)-transduced cell lines **(** **Fig. 5** **and Fig. S2, S3).**

GFP fluorescence, the identifier of reporter-transduced cells, remained unchanged under normoxia, hypoxia or CoCl_2_ treatment and served as an internal control **(** **Fig. 3** **,** **4** **,** **5** **and Fig. S2, S3, S4).** Furthermore, the data presented in **Figure 4** **and Figures S2, S3, S4** clearly demonstrate that the subcellular distribution of the endogenous HIF-1α in wild-type cells is the same as the HIF-1α/FLuc fusion protein in reporter-transduced cells under normal basal growing conditions, CoCl_2_ treatment or hypoxia (2.5%).

### Real-time imaging of HIF-1α stabilization in tumor xenografts

**Figure 6:**
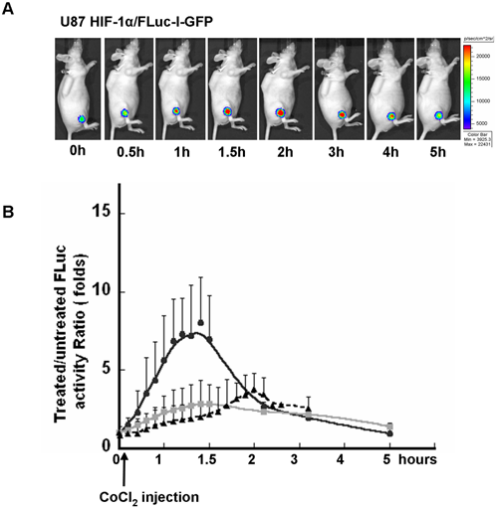
Bioluminescence imaging of HIF-1α/FLuc stabilization *in vivo*. Bioluminescence imaging of HIF-1α/FLuc stabilization in U87/HIF-1α/FLuc xenograft-bearing animals was performed at the indicated time points before and after intraperitoneal injection of CoCl_2_ (60 mg/kg). D-luciferin was delivered by an implanted micro-osmotic pump (A). Graph represents temporal changes in bioluminescence of U87/HIF-1α/Fluc (solid circle), U87/HIF-1α(ΔODDD)/Fluc (grey square), U87/FLuc-IRES-GFP (solid triangle) xenografts treated with CoCl_2_. Data is normalized to the baseline (pre-CoCl_2_) value (B).

To characterize the reporter systems *in vivo*, a series of experiments were performed to image the stabilization of HIF-1α following CoCl_2_ administration and inhibition of prolyl hydroxylases in animals bearing a HIF-1α/FLuc reporter xenograft. Three sets of mice bearing one of three constitutively expressing reporter xenografts were studied: HIF-1α/FLuc, HIF-1α(ΔODDD)/FLuc and FLuciferase (control). After a single intraperitoneal injection of CoCl_2_ (60 mg/kg), a time-dependent increase in tumor bioluminescence was observed in U87/HIF-1α/FLuc xenografts. The maximum bioluminescence intensity, ∼6-fold over pretreatment values, was observed 40–90 min after CoCl_2_ administration. The signal decreased gradually to basal levels over 5 h (**Fig. 6A, B**). In contrast, only a slight increase in bioluminescence after CoCl_2_ injection was detected in mice bearing the U87/HIF-1α(ΔODDD)/FLuc or U87/Fluc control xenografts.

### Assessment of HIF-1α/FLuc and HIF-1α(ΔODDD)/FLuc degradation in different cell lines

**Figure 7:**
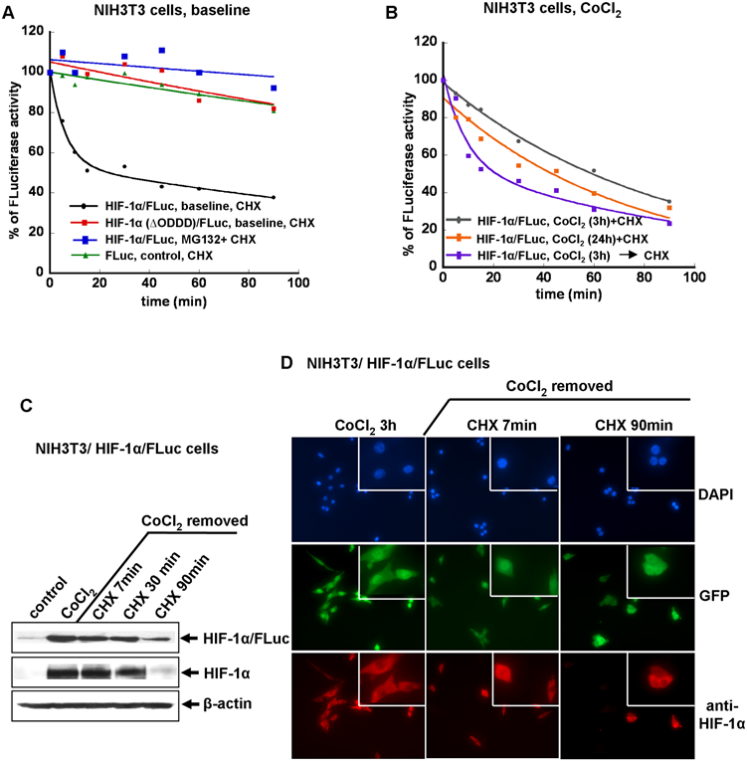
Assessment of HIF-1α/FLuc degradation in NIH3T3 cells under different conditions. The degradation rate of the reporter proteins (HIF-1α/FLuc, HIF-1α(ΔODDD)/FLuc and FLuc) was determined by sequential bioluminescence measurements following addition of a protein synthesis inhibitor (CHX 100 µg/ml) to the medium. The data were fitted to either a single or double exponential equation to estimate the protein degradation rate (half-life). The graphic plots and fitted curves are representative experiments (A and B); NIH3T3 cells lysates were used for immunoblotting with antibodies to HIF-1α to assess HIF-1α/FLuc fusion protein and endogenous HIF-1α protein degradation after CoCl_2_ - containing media was removed and replaced with CHX containing media for 0, 7 and 90 min (C). Under the same conditions as that described for panel (C), the cells were analyzed by fluorescence imaging using the same procedure, described in the legend to Figure 3.

**Figure 8:**
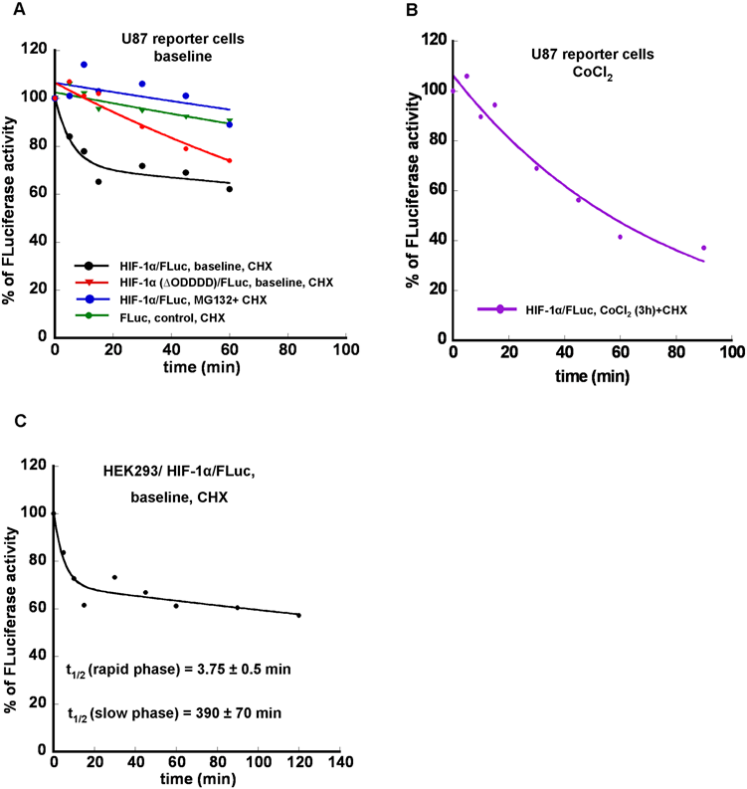
Assessment of HIF-1α/FLuc degradation in U87 and HEK293 cells under different conditions. The degradation rate of the reporter proteins (HIF-1α/FLuc, HIF-1α(ΔODDD)/FLuc and FLuc) was determined by sequential bioluminescence measurements following addition of a protein synthesis inhibitor (CHX,100 µg/ml) to the medium. The data were fitted to either a single or double exponential equation to estimate the protein degradation rate (half-life). The graphic plots show a representative set of experiments (A, B and C).

To assess pVHL-ODDD-dependent degradation of HIF-1α/FLuc in NIH3T3 and U87 reporter cells, the time-course and the degradation rate of the fusion proteins (HIF-1α/FLuc and HIF-1α(ΔODDD)/FLuc), as well as FLuc alone was determined by sequential bioluminescence measurements following the addition of a protein synthesis inhibitor (cycloheximide, 100 µg/ml) to the incubation medium (**Fig. 7** **and** **8**). Degradation of the HIF-1α/FLuc fusion protein in reporter-transduced NIH3T3 and U87 cells under normoxic conditions had a bi-exponential clearance, readily separated into rapid (mean t_1/2_ 4–6 min) and slow (mean t_1/2_ ∼200 min) kinetic phase components (**Fig. 7A** **and** **8A** **;** **Tables 1** **and** **2**). A similar bi-exponential clearance profile of HIF-1α/FLuc was observed in HEK293 reporter cells (**Fig. 8C**). In contrast, only a single exponential, reflecting a slow clearance phase (mean t_1/2_ ∼160 min) was identified in both NIH3T3 and U87 cells transduced with the HIF-1α(ΔODDD)/FLuc fusion construct. Similarly, only a single, very slow clearance component (mean t_1/2_ ∼400 min) was identified for the constitutively expressed, native FLuc protein under normoxic conditions in NIH3T3/FLuc and U87/FLuc reporter cells (**Fig. 7A** **and** **8A** **;** **Tables 1** **and** **2**).

**Table 1:** Determination of HIF-1α/FLuc half-life in NIH3T3 cells.

REPORTER SYSTEM	CONDITIONS	T_1/2_ RAPID PHASE (MINUTES)	T_1/2_ SLOW PHASE (MINUTES)	R MEAN	N
HIF-1α/FLuc	baseline	6±1.1	217±53	0.99	4
HIF-1α/FLuc	*CoCl_2_ (3h)→CHX	8±2	65±10	0.98	4
HIF-1α/FLuc	CoCl_2_ (3h)+CHX	NF	63±29	0.99	4
HIF-1α/FLuc	CoCl_2_ (24h)+CHX	NF	42±13	0.98	3
HIF-1α(ΔODDD)/FLuc	baseline	NF	168±80	0.94	6
HIF-1α/FLuc	MG132+CHX	NF	742±300	0.46	3
FLuc	baseline	NF	358±113	0.84	4

The mean HIF-1α/FLuc, HIF-1α(ΔODDD)/FLuc and FLuc protein half-lives were calculated based on 3–6 independent experiments.

* CoCl_2_ was removed from medium before CHX addition; NF- not found.

**Table 2:** Determination of HIF-1α/FLuc half-life in U87 cells.

REPORTER SYSTEM	CONDITIONS	T_1/2_ RAPID PHASE (MINUTES)	T_1/2_ SLOW PHASE (MINUTES)	R MEAN	N
HIF-1α/FLuc	baseline	3.5±0.8	276±141	0.93	4
HIF-1α/FLuc	CoCl_2_ (3h)+CHX	NF	51±16	0.97	3
HIF-1α(ΔODDD)/FLuc	baseline	NF	148±48	0.91	3
HIF-1α/FLuc	MG132+CHX	NF	302±30	0.83	3
FLuc	baseline	NF	414±105	0.90	4

The mean HIF-1α/FLuc, HIF-1α(ΔODDD)/FLuc and FLuc protein half-lives was calculated based on 3–6 independent experiments.

The presence of CoCl_2_ (100 µM) together with cycloheximide (100 µg/ml) in the medium resulted in a loss of the rapid clearance component in both NIH3T3/HIF-1α/FLuc (**Fig. 7B** **;** **Table 1**) and U87/HIF-1α/FLuc cells (**Fig. 8B** **;** **Table 2**). The clearance profile could be described by a single exponential with a mean half-time of ∼50 min after a 3 h or a 24 h exposure to CoCl_2_. The precise mechanism for regulating the HIF-1α levels is still not clear; both increased degradation [29] and decreased production of the protein have been proposed [30], [31]. Reappearance of the bi-exponential clearance of the HIF-1α/FLuc fusion protein, reflecting rapid and slow degradative phase, was again observed after removal of CoCl_2_ from the incubation medium in NIH3T3 reporter-transduced cells (**Fig. 7B** **;** **Table 2**). The presence of residual, non-degraded HIF-1α/FLuc protein after 90 min of protein synthesis inhibition was demonstrated by Western blotting (**Fig. 7C**), and comparable levels and rates of degradation were observed by immunoblot for endogenous HIF-1α and HIF-1α/FLuc. Thus, partial degradation of the fusion protein with a persistence of a functional Firefly luciferase component is unlikely. To demonstrate involvement of the proteasome in the degradation of the HIF-1α, cell lines bearing the HIF-1α/FLuc reporter cells were pretreated with a proteasome inhibitor, MG132 (10 µM for 3 h), followed by the addition of cycloheximide to the medium. MG132 prevented HIF-1α/FLuc protein degradation in all cell lines (**Fig. 7A** **and** **8A** **;** **Tables 1** **and** **2**).

To determine if the observed rapid and slow clearance components reflect different rates of cytoplasmic and nuclear HIF-1α/FLuc protein degradation, NIH3T3/HIF-1α/FLuc reporter cells were analyzed by immunofluorescence microscopy **(** **Fig. 7D**). Reporter cells were pretreated with CoCl_2_ for 3 h, followed by its removal from the media and the addition of cycloheximide for 0, 7 and 90 min. HIF-1α/FLuc distribution within cells at 7 min after addition of cycloheximide showed slightly more nuclear intensity and a few cells visualized by DAPI staining and GFP expression were not visualized by anti-HIF-1α fluorescent antibody. By 90 minutes, approximately half the reporter cells were not visualized by anti-HIF-1α immunofluorescence **(** **Fig. 7D**), suggesting that some cells clear the HIF-1α/FLuc fusion protein more rapidly then other cells. GFP immunofluorescence remained unchanged under cycloheximide treatment and was used as an internal control to identify reporter-transduced cells **(** **Fig. 7D**).

## Discussion

The two HIF-1α chimeric reporter systems that were developed in this study provide an opportunity to investigate HIF-1α stabilization/degradation process in different cell lines, both in culture and in xenografts. Several groups have studied and employed the fusion between the HIF-1α-ODD domain and Firefly Luciferase. The resulting fusion protein (ODD-Luc) is responsive to hypoxia and hypoxia mimetics in live cells and can be used for imaging HIF-1 oxygen/VHL-regulated activity in real-time under different conditions [32], [33], [34]. A mouse that ubiquitously expresses the ODD-Luc reporter has been successfully used to study the action of small molecule inhibitors of HIF prolyl hydroxylase activity [35]. However, the ODD-Luc reporter is useful only for studying the O_2_-ODD-VHL-dependent mechanism of regulating HIF-1α expression. HIF-1α protein levels are also regulated by oxygen-independent mechanisms that reflect genetic alterations in signaling pathways or regulatory factors, and result in constitutive high levels of HIF-1α and HIF-1 transcriptional activity [36], [37]. Two PAS domains, A and B, of the HIF-1α subunit in the N-terminal region frequently mediate protein-protein interactions. One or both of the HIF-1α PAS domains have been functionally implicated in heterodimer formation, nuclear translocation and HIF-1α stabilization via HSP90 association [38]. The C-terminal part of the HIF-1 α is involved in protein transactivation. That is why the presence of the entire length of HIF-1α in reporter protein is essential for understanding HIF-1α biology and regulation of HIF-1α stability, since this occurs at multiple levels and involves more than the ODD domain.

A comparison between HIF-1α/Fluc and HIF-1α(ΔODDD)/Fluc expression levels, as measured by BLI, demonstrated important differences between non-tumorigenic NIH3T3 and HEK293 reporter cells and tumorigenic PTEN-defective U87 reporter cells. Non-tumorigenic NIH3T3 and HEK293 cells had low basal normoxic levels of the HIF-1α/FLuc expression that were readily detectable by BLI, but not by immunoblotting. The higher sensitivity of the bioluminescence reporter in comparison to the immunoblot readout in living cells provides the ability to detect low basal levels of HIF-1α expression without exposing cells to hypoxia or hypoxia-mimetics agents. In contrast, U87 reporter cells had high basal levels of HIF-1α/FLuc expression. Similar results have been shown for other cancer cells lines using an immunoblotting assessment of endogenous HIF-1α. For example, HIF-1α overexpression under standard growth conditions was detected by immunoblot in metastatic breast cancer MDA-MB-231 cells and DU145 prostate cancer cell lines [39]. Interestingly, other cells (including MCF-7, HT-29 colon, MiaPaCa pancreatic, A549 lung, and BX-PC3 prostate cancer cells) do not show measurable HIF-1α levels under normoxia [39]. In addition, our reporter systems demonstrated that various cell lines responded differently to inhibition of a prolyl hydroxylases, either by hypoxia or CoCl_2_. The reporter-transduced U87 cells showed a weaker response to CoCl_2_ and hypoxia, relative to normoxic baseline values, when compared to that observed in NIH3T3 reporter-transduced cells.

Further validation of the reporter systems was obtained with NIH3T3 and U87 cells bearing a mutated HIF-1α(ΔODDD)/Fluc fusion gene. Both cell lines demonstrated similar high levels of reporter expression (BLI and immunoblot), reflecting the effect of ODDD deletion on the stability of the fusion protein [35]. In addition, we demonstrated a significant BLI response *in vivo* when animals bearing U87/HIF-1α/FLuc xenografts were given an i.p injection of CoCl_2_, but not in animals bearing U87/HIF-1α(ΔODDD)/FLuc or native FLuc expressing (control) xenografts.

The immunofluorescence analysis of the HIF-1α/FLuc subcellular localization and trafficking in reporter-transduced cell lines compared well with that of endogenous HIF-1α in wild-type cells. However, significant differences were observed between NIH3T3, HEK293 and U87 cells. These observations suggest a difference in cytoplasm-nuclear trafficking of the HIF-1α and its protein fusion between normal and tumor cells. It was previously shown under normal oxygen tension that pVHL is engaged in a constitutive shuttling between the nucleus and cytoplasm [13], [40]. Furthermore, it is generally accepted that subcellular localization of the HIF-α subunit in mammalian cells depends on the tumor suppressor factor pVHL, which mediates ubiquitination of HIF-1α. However, it has not been fully established whether proteasomal degradation of HIF-1α occurs in the nucleus, in the cytoplasm, or if it can take place in both compartments. Prior reports are somewhat contradictory. According to some reports [41], [42] and the results of our immunofluorescence microscopic studies, the HIF-1α (and HIF-1α/FLuc) translocation to the nucleus appears to be constitutive and independent of oxygen tension, pVHL status and the presence of a functional ODD domain in HIF-1α [13], [14]. In many cell lines HIF-1α-pVHL interaction is abrogated in response to hypoxia or hypoxia-mimetic agents, and HIF-1α accumulates in the nucleus [43], [44], [45]. This was clearly demonstrated for the HIF-1α/FLuc fusion in U87 and HEK293 reporter cells, and for endogenous HIF-1α in U87 and HEK293 wild-type cells, and may be occurring to a lesser degree in NIH3T3 cells as well. The presence of HIF-1α/FLuc and HIF-1α in the cytoplasm and nucleus of NIH3T3 reporter and wild-type cells exposed to CoCl_2_ and hypoxia indicates continuous shuttling of the protein between the nucleus and the cytoplasm. We speculate that the degradation of HIF-1α in this cell type can take place in both compartments at different rates, possibly due to a residual hydroxylation of HIF-1α or to other non-oxygen-dependent process.

The predominant nuclear localization of HIF-1α/FLuc and HIF-1α in U87 cells under normoxic conditions indicates that these proteins are either imported or sequestered to the nucleus in U87 reporter cells to a greater extent than that in NIH3T3 cells, or that their nucleus-to-cytoplasm shuttling of HIF-1α is reduced in U87 compared to NIH3T3 cells. This is consistent with the 8–10 fold difference observed in bioluminescence between the two cell lines under normoxic conditions. Reduced pVHL trafficking was recently demonstrated in clear cell renal cell carcinoma, and was associated with strong nuclear localization of HIF-1α and shorter patient survival [46]. Although the mechanism of HIF-1α shuttling between nucleus and cytoplasm is poorly understood, it is becoming evident, that HIF-1α subcellular distribution and compartmentalization of HIF-1α degradation are regulated in a cell-specific manner [47] and that there are significant differences between normal cells and cancer cells. This may reflect pVHL malfunction in cancer cells or activation of the other transduction pathways in cancer cells.

For example, activation of the PI3K-AKT/mTOR pathway impacts on HIF-1 through an increase in HIF-1α protein levels [37], caused by augmentation of the protein translation [9],[48] or by stabilization of the protein [36]. In addition, it was reported that activation of the PI3K-AKT/mTOR pathway also causes a major increase in nuclear localization of HIF-α/Sima from *Drosophila melanogaster,* which is a functional homologue of mammalian HIF-1α [49]. U87 human glioma cells are PTEN-negative with increased PI3K-AKT/mTOR pathway activity [24], [50]. The difference in nuclear localization of HIF-α/Fluc and wild-type HIF-α in this cell line compare with a normal cells is a new observation and requires further investigation.

Deletion of the ODDD in the HIF-1α/Fluc fusion protein prevents hydroxylation by prolyl-hydroxylases and abrogates HIF-1α(ODDD)–pVHL interaction. The HIF-1α(ODDD)/Fluc fusion was shown to have markedly reduced nucleus-to-cytoplasm shuttling in NIH3T3 and U87 reporter cells, and increased accumulation of the mutant fusion protein in the nucleus. This led to the generation of high bioluminescence signals from the stabilized protein in the HIF-1α(ODDD)/Fluc reporter cell lines. These results suggest that nuclear export plays a major role in the control of HIF-1α level in cells, adding a new level of complexity to the regulation of this transcriptional factor.

Our immunofluorescence observations prompted us to study the dynamics of HIF-1α/FLuc protein degradation in greater detail. The question being addressed is whether the difference in the level of HIF-1α/FLuc expression under baseline normoxic conditions, as well as the ability of this fusion protein to accumulate following exposure to CoCl_2_ and to moderate hypoxia, is due to a difference in the pVHL-ODDD-dependent degradation process of HIF-1α/FLuc in different cell lines. A bi-exponential bioluminescence profile of HIF-1α/FLuc protein degradation was observed in NIH3T3, HEK293 and U87 reporter cells, indicating that both “rapid” and “slow” clearance mechanisms were operative. The half-time of the rapid clearance in these cells was ∼3–6 min and consistent with the currently accepted half-life of HIF-1α (∼5 min) under normal non-hypoxic conditions [51], [52], [53]. Since the rapid phase HIF-1α/FLuc degradation was abolished by CoCl_2_ treatment and by deletion of the oxygen-dependent degradation domain from HIF-1α in NIH3T3 and U87 reporter cells, it is likely that the rapid clearance component reflects the dynamics of the oxygen-dependent pathway of HIF-1α degradation in these cells. A second, slow clearance component of the HIF-1α/FLuc degradation under normoxic conditions in these cell lines was also revealed in our kinetic analysis of the bioluminescence clearance profile. This component does not reflect degradation of native FLuc protein since immunoblots of cells taken 7, 30 and 90 min after addition of cycloheximide in the clearance studies showed the presence of both non-degraded HIF-1α/FLuc and non-degraded HIF-1α bands.

The dynamic profile and kinetic analysis of the HIF-1α/FLuc degradation in NIH3T3, HEK293 and U87 cells suggests that the rapid and slow components of degradation are compartmentalized. To determine, whether the observed kinetic and anatomic compartmentalization in the cell are related, we imaged the changes in subcellular distribution during cycloheximide-induced clearance of the HIF-1α/FLuc protein in NIH3T3 reporter-transduced cells by immunofluorescence imaging. Some of reporter cells cleared their fluorescence (both cytoplasmic and nuclear) more quickly than others. This observation suggests that the observed kinetic compartmentalization may reflect a heterogeneity and difference in HIF-1α/FLuc protein degradation rate within two or more functionally different populations of NIH3T3/HIF-1α/FLuc cells. Despite a similar bi-phasic degradation profile of HIF-1α/FLuc in NIH3T3 and U87 reporter cells, the basal expression level and subcellular localization of the fusion protein in these cell lines is very different. This suggests the involvement of other regulatory mechanisms, which may account for differences in the spacial distribution and stabilization of HIF-1α in cells with different genetic backgrounds.

The expression of HIF-1α protein is modulated through different pathways that alter HIF-1-dependent transcriptional activity and makes the HIF-1 transcription factor an attractive target for new drug development [4], [54], [55]. Several examples of HIF-1 inhibitors that target pathways associated with HIF-1 activation have been described [55]. For example, a number of anticancer drugs have been shown to inhibit HIF-1, but none of these drugs target HIF-1 directly and specifically [4], [54], [56], [57], [58], [59], [60], [61], [62], [63],[64], [65], [66], [63]. Some of these inhibitors are associated with signaling pathways that include mTOR [36], [67], AKT [68], Her2/Neu [9]. However, most inhibitors affect multiple signaling pathways and only indirectly target the HIF-1 transcription factor. Therefore, the ability to identify, visualize and validate changes in the dynamics and stability of the HIF-1α protein is likely to be useful as a pharmacodynamic end point and read-out of inhibition in the development of new anticancer drugs that target HIF-1α in the future.

In summary, the introduction of a *HIF-1α/FLuc* fusion gene into different cell lines allowed us: 1) to study the time-dependent accumulation and degradation of the fusion protein with greater sensitivity and to better quantify the rate of degradation using BLI, compared to that obtained in previous studies based on immunoblot assays; 2) to better assess the differences in subcellular localization and clearance of the fusion protein in normal and tumor cells using immunofluorescence imaging, providing spatial information to better understand HIF-1α trafficking and regulation in different cells; 3) to image HIF-1α/FLuc fusion protein stabilization and accumulation over time in animals bearing reporter-transduced xenografts. Finally, the possibility to visualize the modulation of HIF-1α using the chimeric reporter systems developed in the current study, in combination with transcriptional reporters based on the binding of an active HIF-1 complex to hypoxia-response elements (HRE) which drive reporter genes [28], [69], [70], [71], [72], [73], provides the opportunity to utilize them as tools for high-throughput screening of compounds affecting HIF-1 activity, both *in vitro* and *in vivo*.

## Supporting Information

Figure S1 FACS analysis of reporter-transduced cells. GFP expression in developed reporter cell lines was used for FACS sorting and transduction normalization.
(0.91 MB TIF)

Figure S2 Immunofluorescence analysis of HIF-1α and its fusions expression in NIH3T3 cells. The NIH3T3/HIF-1α/Fluc, NIH3T3/HIF-1α(ΔODDD)/Fluc and NIH3T3 non-tansduced cells were cultured under normoxia and hypoxia for 6 h. Cells were prepared for immunofluorescence as described in Materials and Methods and then were incubated with the anti-HIF-1α antibody followed by a Alexa-568-conjugated secondary antibody. Cells were also visualized for GFP expression. Fluorescence images were acquired at ×40 magnification. All panels represent the magnified images after adjusted contrast and brightness to visualize the subcellular protein localization.
(4.01 MB TIF)

Figure S3 Immunofluorescence analysis of HIF-1α and its fusions expression in U87 cells. The U87/HIF-1α/Fluc, U87/HIF-1α(ΔODDD)/Fluc and U87 non-tansduced cells were cultured under normoxia and hypoxia for 6 h. Cells were prepared for immunofluorescence as described in Materials and Methods and then were incubated with the anti-HIF-1α antibody followed by a Alexa-568-conjugated secondary antibody. Cells were also visualized for GFP expression. Fluorescence images were acquired at ×40 magnification. All panels represent the magnified images after adjusted contrast and brightness to visualize the subcellular protein localization.
(3.46 MB TIF)

Figure S4 Immunofluorescence analysis of HIF-1α and HIF-1α/FLuc expression in HEK293 cells. The reporter-transuced HEK293/HIF-1α/Fluc and non-transduced HEK 293 cells were cultured under normoxia or hypoxia for 6 h. Cells were prepared for immunofluorescence as described in Materials and Methods and then were incubated with the anti-HIF-1α antibody followed by a Alexa-568-conjugated secondary antibody. The reporter cells were also visualized for GFP expression. Fluorescence images were acquired at ×40 magnification. All panels represent the magnified images after adjusted contrast and brightness to visualize the subcellular protein localization.
(3.10 MB TIF)
