# Supplementary figures and images for: Real-Time Imaging of HIF-1α Stabilization and Degradation
Source: PLoS One. 2009 Apr 4;4(4):e5077. doi: 10.1371/journal.pone.0005077 (PMC2660410; doi:10.1371/journal.pone.0005077)

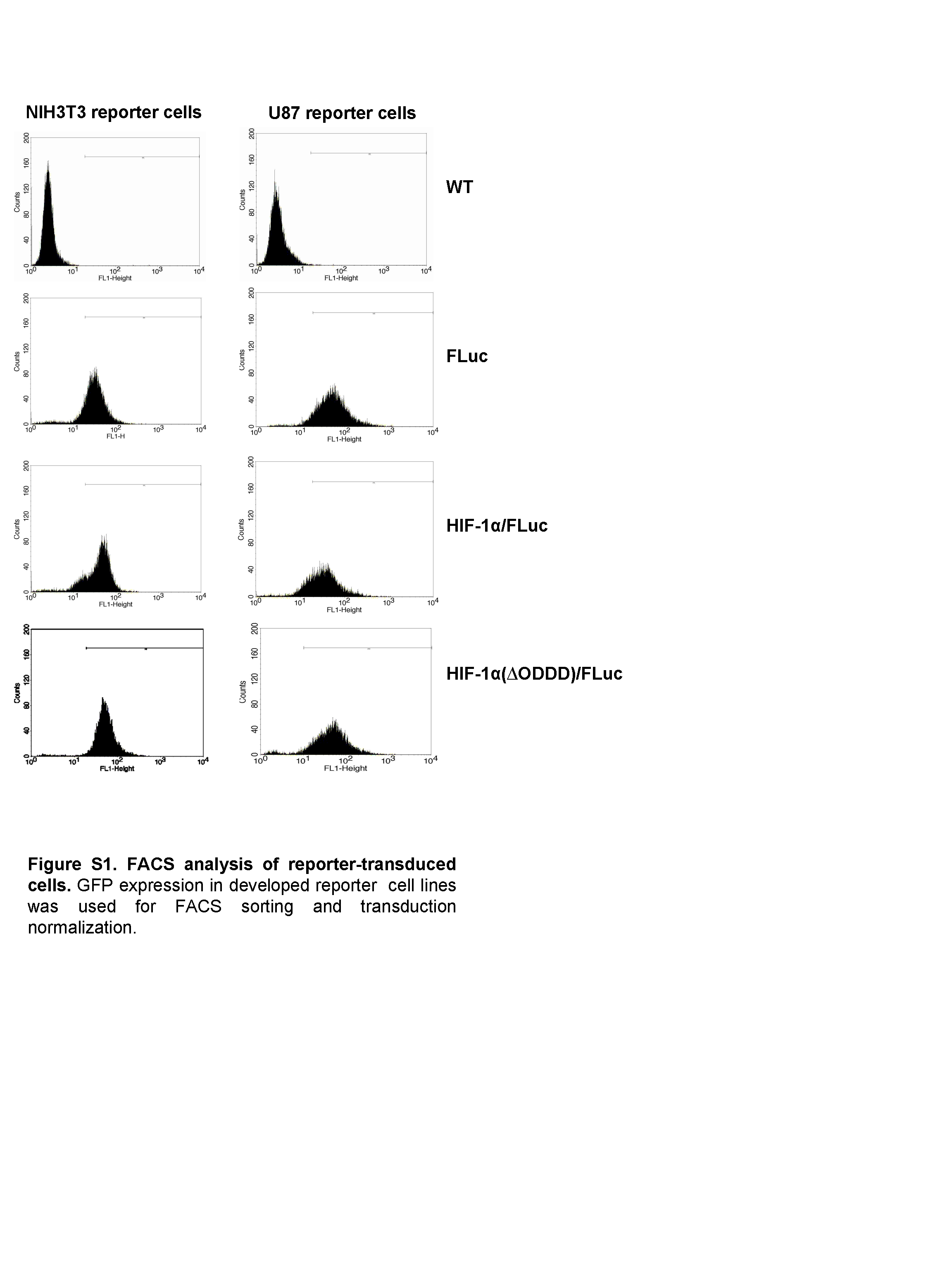

Supplement: Figure S1 — FACS analysis of reporter-transduced cells. GFP expression in developed reporter cell lines was used for FACS sorting and transduction normalization. (0.91 MB TIF) [file pone.0005077.s001.tif]

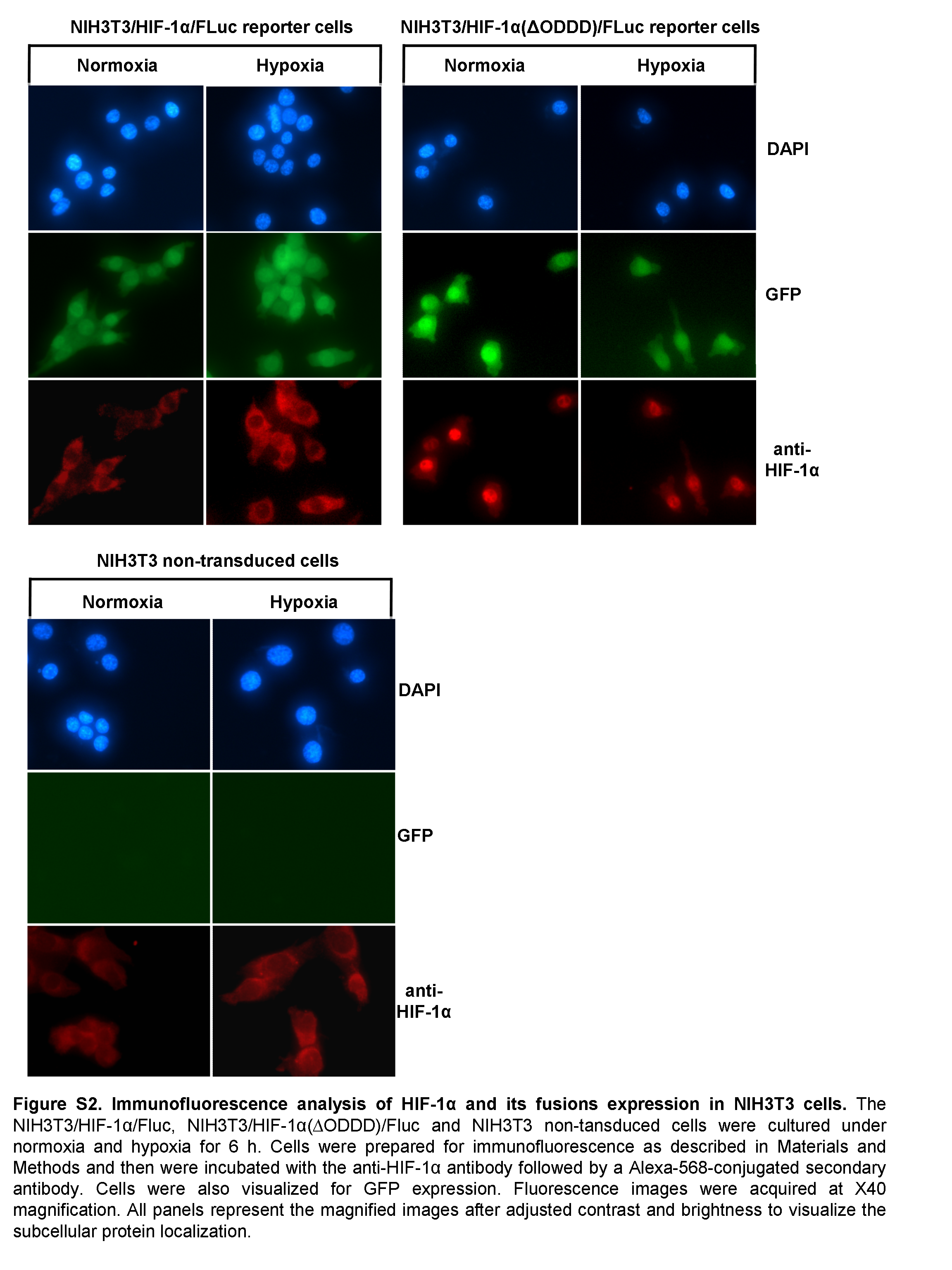

Supplement: Figure S2 — Immunofluorescence analysis of HIF-1α and its fusions expression in NIH3T3 cells. The NIH3T3/HIF-1α/Fluc, NIH3T3/HIF-1α(ΔODDD)/Fluc and NIH3T3 non-tansduced cells were cultured under normoxia and hypoxia for 6 h. Cells were prepared for immunofluorescence as described in Materials and Methods and then were incubated with the anti-HIF-1α antibody followed by a Alexa-568-conjugated secondary antibody. Cells were also visualized for GFP expression. Fluorescence images were acquired at ×40 magnification. All panels represent the magnified images after adjusted contrast and brightness to visualize the subcellular protein localization. (4.01 MB TIF) [file pone.0005077.s002.tif]

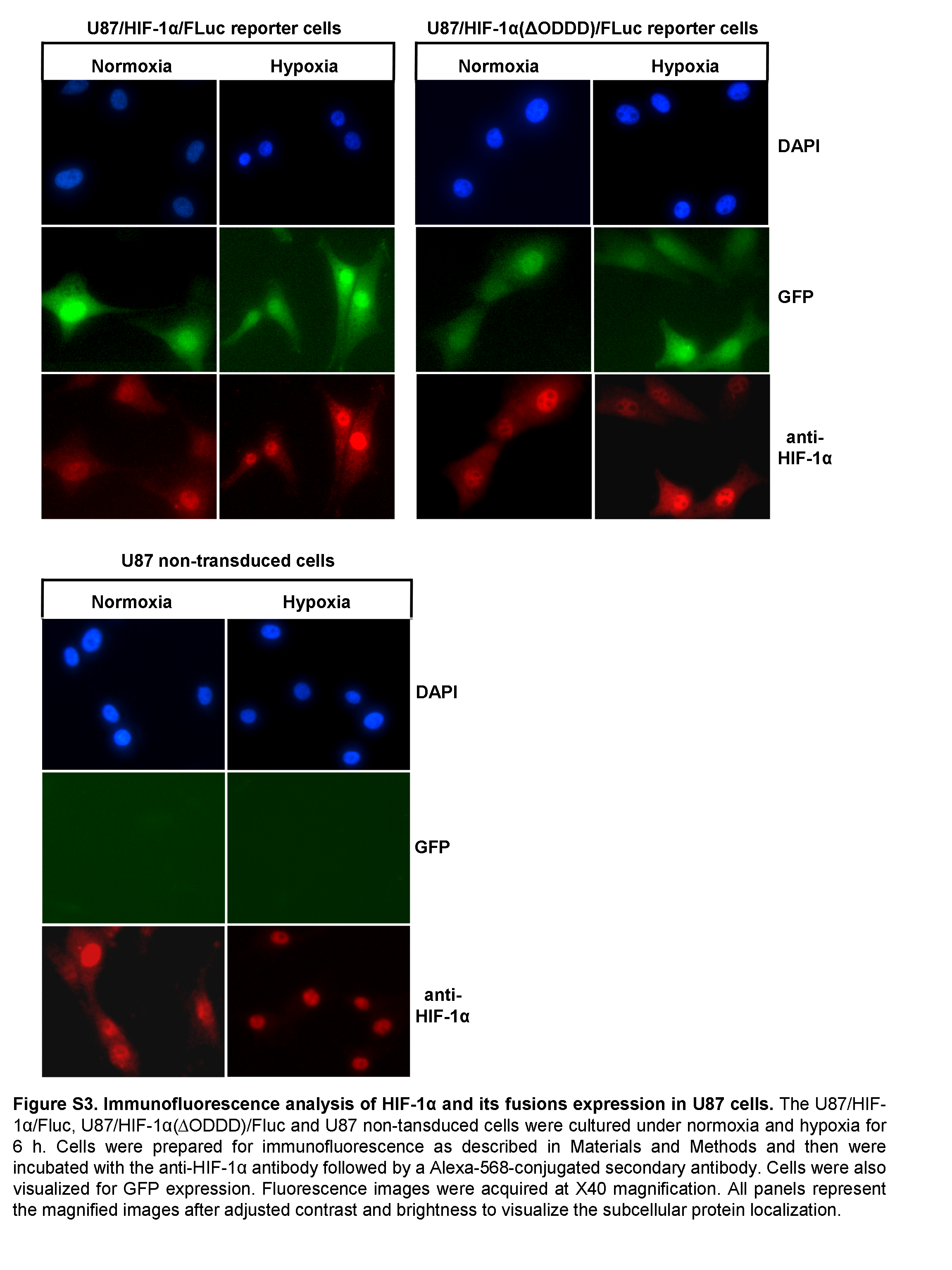

Supplement: Figure S3 — Immunofluorescence analysis of HIF-1α and its fusions expression in U87 cells. The U87/HIF-1α/Fluc, U87/HIF-1α(ΔODDD)/Fluc and U87 non-tansduced cells were cultured under normoxia and hypoxia for 6 h. Cells were prepared for immunofluorescence as described in Materials and Methods and then were incubated with the anti-HIF-1α antibody followed by a Alexa-568-conjugated secondary antibody. Cells were also visualized for GFP expression. Fluorescence images were acquired at ×40 magnification. All panels represent the magnified images after adjusted contrast and brightness to visualize the subcellular protein localization. (3.46 MB TIF) [file pone.0005077.s003.tif]

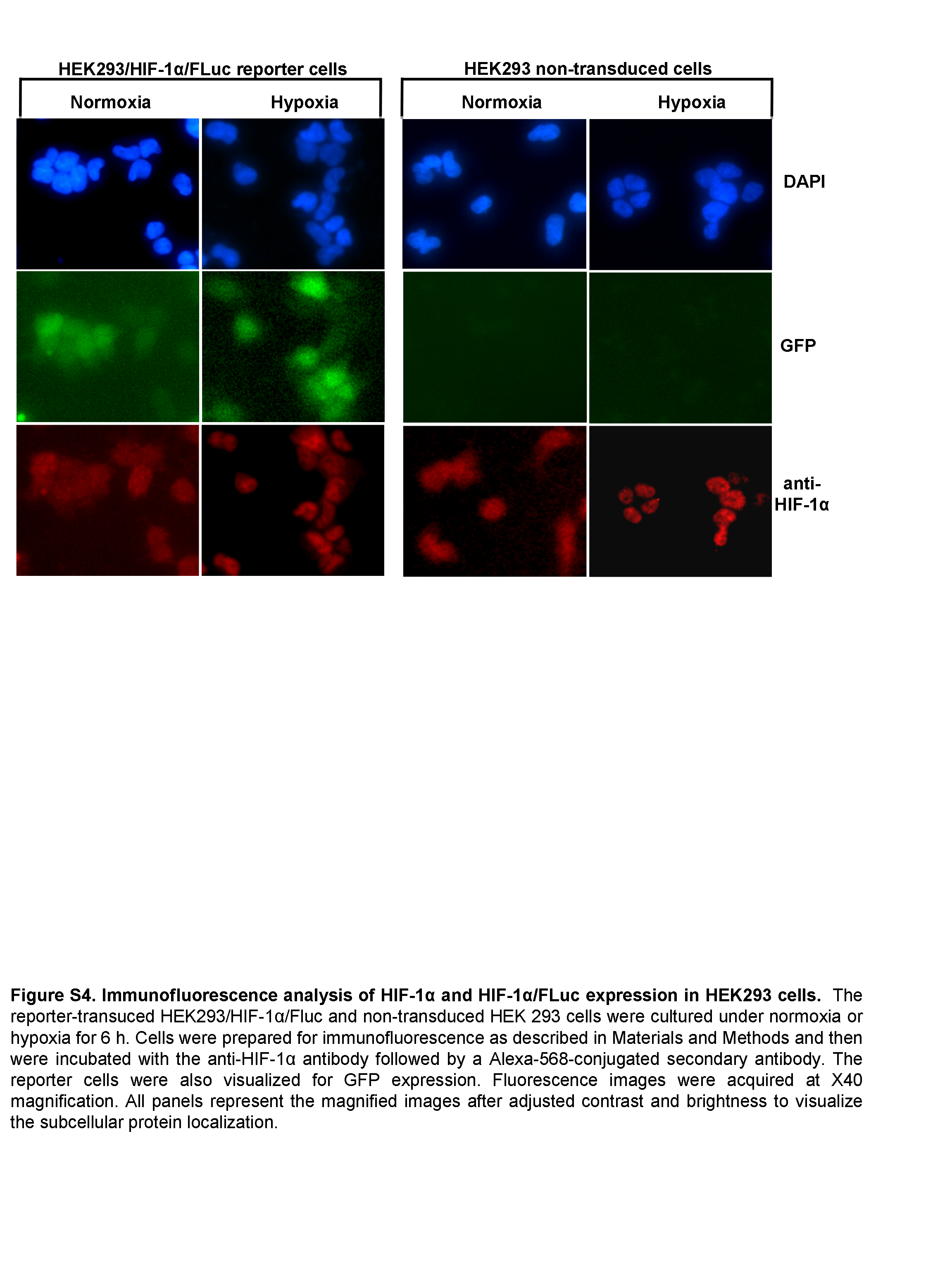

Supplement: Figure S4 — Immunofluorescence analysis of HIF-1α and HIF-1α/FLuc expression in HEK293 cells. The reporter-transuced HEK293/HIF-1α/Fluc and non-transduced HEK 293 cells were cultured under normoxia or hypoxia for 6 h. Cells were prepared for immunofluorescence as described in Materials and Methods and then were incubated with the anti-HIF-1α antibody followed by a Alexa-568-conjugated secondary antibody. The reporter cells were also visualized for GFP expression. Fluorescence images were acquired at ×40 magnification. All panels represent the magnified images after adjusted contrast and brightness to visualize the subcellular protein localization. (3.10 MB TIF) [file pone.0005077.s004.tif]
